# Biomechanical Evaluation of Intervertebral Fusion Process After Anterior Cervical Discectomy and Fusion: A Finite Element Study

**DOI:** 10.3389/fbioe.2022.842382

**Published:** 2022-03-17

**Authors:** Yi-Wei Shen, Yi Yang, Hao Liu, Yue Qiu, Ming Li, Li-Tai Ma, Fang-Ji Gan

**Affiliations:** ^1^ Department of Orthopedics, Orthopedic Research Institute, West China Hospital, Sichuan University, Chengdu, China; ^2^ West China Biomedical Big Data Center, West China Hospital, Sichuan University, Chengdu, China; ^3^ Department of Measurement and Control Technology and Instrument, Sichuan University, Chengdu, China

**Keywords:** cervical spine, finite element analysis, anterior cervical discectomy and fusion, interbody fusion cage, biomechanics

## Abstract

**Introduction:** Anterior cervical discectomy and fusion (ACDF) is a widely accepted surgical procedure in the treatment of cervical radiculopathy and myelopathy. A solid interbody fusion is of critical significance in achieving satisfactory outcomes after ACDF. However, the current radiographic techniques to determine the degree of fusion are inaccurate and radiative. Several animal experiments suggested that the mechanical load on the spinal instrumentation could reflect the fusion process and evaluated the stability of implant. This study aims to investigate the biomechanical changes during the fusion process and explore the feasibility of reflecting the fusion status after ACDF through the load changes borne by the interbody fusion cage.

**Methods:** The computed tomography (CT) scans preoperatively, immediately after surgery, at 3 months, and 6 months follow-up of patients who underwent ACDF at C5/6 were used to construct the C2–C7 finite element (FE) models representing different courses of fusion stages. A 75-N follower load with 1.0-Nm moments was applied to the top of C2 vertebra in the models to simulate flexion, extension, lateral bending, and axial rotation with the C7 vertebra fixed. The Von Mises stress at the surfaces of instrumentation and the adjacent intervertebral disc and force at the facet joints were analyzed.

**Results:** The facet contact force at C5/6 suggested a significantly stepwise reduction as the fusion proceeded while the intradiscal pressure and facet contact force of adjacent levels changed slightly. The stress on the surfaces of titanium plate and screws significantly decreased at 3 and 6 months follow-up. A markedly changed stress distribution in extension among three models was noted in different fusion stages. After solid fusion is achieved, the stress was more uniformly distributed interbody fusion in all loading conditions.

**Conclusions:** Through a follow-up study of 6 months, the stress on the surfaces of cervical instrumentation remarkably decreased in all loading conditions. After solid intervertebral fusion formed, the stress distributions on the surfaces of interbody cage and screws were more uniform. The stress distribution in extension altered significantly in different fusion status. Future studies are needed to develop the interbody fusion device with wireless sensors to achieve longitudinal real-time monitoring of the stress distribution during the course of fusion.

## Introduction

Degenerative cervical spondylosis is a chronic, progressive deterioration of cervical components that occurs in over half of the middle-age population (Irvine et al., 1965; Theodore, 2020). Patients with degenerative cervical spondylosis may present with symptoms of neck pain or neurologic deficits, including radiculopathy, myelopathy, or a combination of these symptoms. Although conservative treatment usually results in abatement of symptoms, surgery intervention is indicated in patients who fail strict conservative management. Since the anterior cervical discectomy and fusion (ACDF) was first reported in 1950s (Smith and Robinson, 1958), it has been one of the most widely used surgical procedures for patients with cervical radiculopathy and myelopathy. Although iliac crest bone graft remains the gold standard in ACDF until now, the interbody cage implant is prevalent among surgeons due to its excellent safety, primary stability, and satisfactory fusion rate without the complications of donor sites (Chong et al., 2015).

A solid interbody fusion is of paramount importance in achieving successful outcomes after ACDF. However, the status of fusion is usually difficult for surgeons to accurately evaluated. Currently, the standard methods for surgeons to examine the degree of interbody fusion included neutral and dynamic x-rays and computed tomography (CT) (Burkus et al., 2017). However, radiographic evidence is an indirect method for fusion assessment and may not adequately reflect the extent of bone healing (Brodsky et al., 1991; Blumenthal and Gill, 1993). Besides, the radiographic images are static observations of the fusion site and cannot provide information about the fusion integrity during various motions of cervical spine. Blumenthal and Gill (1993) found that the plain radiographs resulted in a 20% underestimation of the degree of fusion. Brodsky et al. (1991) noted a decreased accuracy of radiographic techniques in fusion evaluation of multiple spinal segments and emphasized the need for more accurate non-invasive methods to determine the fusion status. In addition, the widely accepted criteria for determining interbody fusion, namely, the formation of continuous bone bridging using CT scans, has obvious deficiencies such as the high cost and potential radiation hazard, especially for patients without obvious symptoms at routinely postoperative follow-up (Walker, 1989; Noordhoek et al., 2019). Thus, an accurate and non-radiative approach to evaluating the degree of fusion after ACDF is needed.

The interbody instrumentation is used to initially function as the load-bearing element to create a stable biomechanical environment during the early stages of bone healing (Kanayama et al., 1997; Benzel and Ferrara, 2001). After a robust interbody fusion is achieved between the adjacent vertebrae, mechanical load originally borne by the instrumentation may transmit to the newly formed bone. Given that different amounts of bone formation at different fusion stages and daily activities in various postures, the mechanical loads exerted on the interbody instrumentation are complex and under dynamic changing after surgery. Therefore, the mechanical loading on the instrumentation may be a promising indicator to evaluate the fusion status after ACDF, which has the potential to advance current diagnostic methods for interbody fusion. Several animal experiments suggested that the mechanical load on the spinal instrumentation may have the potential to reflect the fusion process and evaluated the stability of implant (Takahata et al., 2005; Ferrara et al., 2007; Ferrara et al., 2008; Peterson et al., 2018). However, the inability to precisely measure the mechanical load of interbody implant in human body remains a significant barrier to determine the feasibility of reflecting the status of fusion through load changes on the instrumentation.

Finite element (FE) analysis is an effective method to evaluate the spinal biomechanics after surgery or during bone remodeling process (Palissery et al., 2009; Ganbat et al., 2016; Jin and Park, 2021). Several previous finite element studies have investigated the biomechanical effect of ACDF. Li et al. (2021) investigated the effect of pre-existing degeneration of adjacent segment on its biomechanics after single-level ACDF. The influences of implant selection on the adjacent-level biomechanics after ACDF were also evaluated in several FE studies (Li et al., 2020; Purushothaman et al., 2020; Guo et al., 2021; Zhou et al., 2021). Hua et al. (2020a) and Hua et al. (2020b) compared the biomechanical changes of adjacent segments after one- or two-level anterior cervical surgery with different implants. However, current FE studies predominantly analyzed the effects of ACDF immediately after surgery. The biomechanical changes of adjacent intervertebral disc, facet joint, and stress on the instrumentation during fusion process through longitudinal study paradigm has been poorly reported. In the present study, the cervical spine-implant FE models were developed based on the CT information of a patient who underwent single-level ACDF at pre-operation, immediately post-operation, and 3 and 6 months after surgery, representing non-fusion, incomplete fusion, and complete fusion stages, respectively (Noordhoek et al., 2019; He et al., 2020; Abudouaini et al., 2021). To the best of our knowledge, this is the first study to simulate the biomechanical environment at different fusion stages after ACDF based on the patient’s follow-up data.

## Materials and Methods

The nonlinear three-dimensional FE model of the C2–C7 cervical spine was developed based on the CT images of a patient who underwent ACDF with Zero-P VA (Synthes, Oberdorf, Switzerland) interbody cage at C5/6 without device-related or neurological complications. The preoperative CT images were used to construct the intact model, and the CT scans of immediately post-operation, and 3 and 6 months during follow-up were constructed for analysis. CT scans with a 0.75-mm thickness and a 0.69-mm interval were obtained using a CT scanner (SOMATOM Definition AS+, Siemens, Germany).

### Generation of Cervical Spine Model and Instrument

The DICOM images were imported into the Mimics 19.0 (Materialize Inc., Leuven, Belgium) software to reconstruct the geometric structure of the C2–C7 cervical vertebrae and output STL files. The intervertebral disc geometries were constructed by filling the intervertebral space and connecting the adjacent vertebral bodies. Next, the model was processed using CATIA v5r21 (Dassault Systems Corporation, Velizy-Villacoublay Cedex, France) for denoising, surfacing, and smoothing. The mesh structure was then prepared using Hypermesh 12.0 (Altair, Troy, MI, United States) and imported into ABAQUS 6.9.1 (Dassault Systems Corporation) to set boundary conditions and perform the analysis.

**FIGURE 1:**
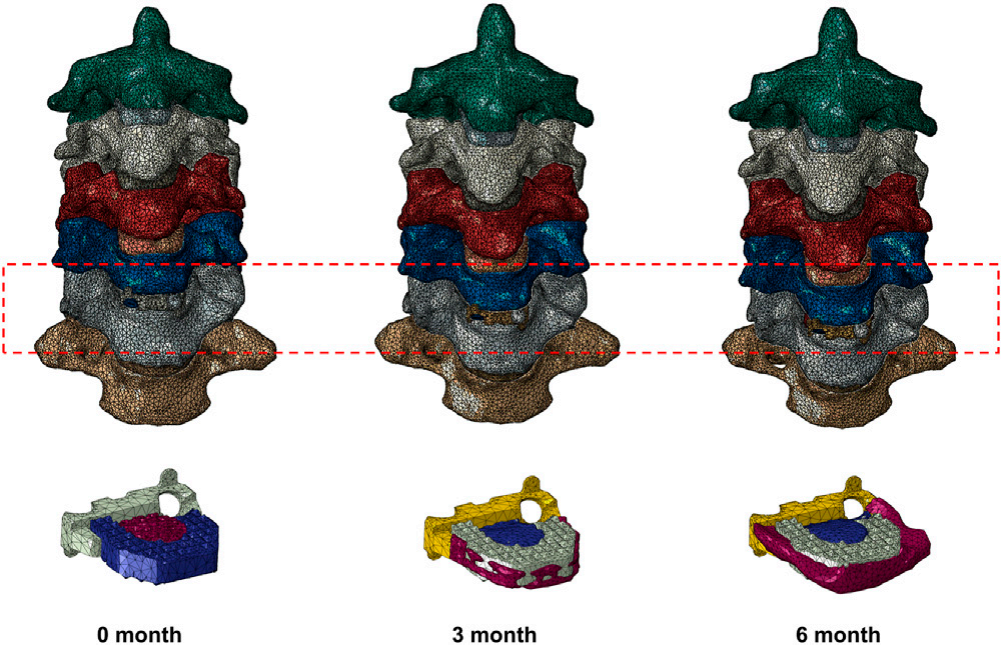
Finite element models of a patient who underwent anterior cervical discectomy and fusion at C5/6 0, 3, and 6 months after surgery with different fusion degrees.

The inner cancellous bone regions of the vertebrae were set as solid elements, surrounded by 0.4-mm-thick shell composed of cortical bone and vertebral endplates (Denozière and Ku, 2006). The intervertebral disc was further divided into the annulus fibrosus and nucleus pulposus with a volume ratio of 6:4 (Denozière and Ku, 2006). Annulus fibers encompassed the ground substance with an inclination to the transverse plane between 15° and 30°, accounting for approximately 19% of the annulus fibrosus volume (Denozière and Ku, 2006; Lee et al., 2011). The facet joint space was simulated to be 0.5 mm, covered by the articular cartilage layer with nonlinear surface-to-surface contact (Rong et al., 2017). The ligamentous complex, including anterior longitudinal ligament (ALL), posterior longitudinal ligament (PLL), ligamentum flavum (LF), interspinous ligament (IL), supraspinous ligament (SL) and capsular ligament (CL), was modeled using tension-only truss elements and attached to the adjacent vertebrae. The interbody fusion device for ACDF was constructed according to the Zero-P VA system, which is composed of the zero-profile titanium plate, the polyetheretherketone (PEEK) cage, and two self-tapping screws in opposite directions. The primary dimensions (width, length, and height) were 13.6, 17.5, and 5 mm, respectively. The self-tapping screws were 16 mm long. The material properties of bone graft and newly formed bone were set as the cortical bone (Figure 1). The material properties and mesh types are listed in Table 1 (Denozière and Ku, 2006; Lee et al., 2011). The number of elements and nodes of the components of cervical spine model are shown in Table 2.

**TABLE 1:** Material properties and mesh types of the cervical finite element model.

	Young modulus (MPa)	Poisson ratio	Element type	Cross sections (mm^2^)
Cortical bone	12,000	0.3	C3D4	—
Cancellous bone	450	0.3	C3D4	—
Annulus fibrosus substance	4.2	0.49	C3D4	—
Annulus fibers	110	0.3	T3D2	—
Nucleus pulposus	1.0	0.49	C3D4	—
Facet joint cartilage	10.4	0.4	C3D4	—
ALL	10	0.3	T3D2	6.0
PLL	10	0.3	T3D2	5.0
CL	10	0.3	T3D2	46.0
LF	1.5	0.3	T3D2	5.0
IL	1.5	0.3	T3D2	10.0
SL	1.5	0.3	T3D2	5.0
Titanium plate	110,000	0.3	C3D4	—
Screws	110,000	0.3	C3D4	—
PEEK	3,600	0.3	C3D4	—

ALL, anterior longitudinal ligament; PLL, posterior longitudinal ligament; CL, capsular ligament; LF, ligamentum flavum; IL, interspinous ligament; SL, supraspinous ligament; C3D4, tetrahedron; T3D2, truss, tension only.

**TABLE 2:** The number of elements and nodes for the cervical spine model.

	Element	Node
C2	71671	14682
C3	54182	11394
C4	67518	13933
C5	65195	14091
C6	85424	18180
C7	72439	14999
C2/3	9675	2780
C3/4	10683	2992
C4/5	9985	2801
C5/6	9096	16124
C6/7	12294	3464
ALL	166	182
PLL	175	195
CL	150	200
LF	122	152
IL	83	98
SL	280	290

ALL, anterior longitudinal ligament; PLL, posterior longitudinal ligament; CL, capsular ligament; LF, ligamentum flavum; IL, interspinous ligament; SL, supraspinous ligament.

### Boundary and Loading Conditions

A tie connection was assigned between the intervertebral discs and adjacent vertebral bodies, and between the insertion of ligaments to bone. The facet joint was built as a nonlinear three-dimensional contact problem using surface-to-surface elements. Frictionless contact was defined between the articular surfaces of the facet joints. The bone that grafted into the central cavity of cage was defined as frictionless (Completo et al., 2015). The superior endplate of C6 was set to connect with central grafted bone. The interactions between the central grafted bone and inferior endplate of C5 were defined as non-connection in postoperative model, partial connection with formed convex surfaces in the 3-month model, and complete connection in the 6-month model. A tie connection was assigned between the newly formed bone outside the cage and endplates. The interaction between the superior and inferior surfaces of the interbody implant and the relevant endplate surfaces was assigned with nonbonded contact formulation (Galbusera et al., 2008), with a contact friction coefficient of 0.1 between anterior titanium plate and endplates and 0.5 between posterior PEEK cage and endplates. A tie constraint was applied to the screw–vertebrae interface to represent sufficient osseointegration. Shared nodes at the screw-plate interfaces were used, thus not allowing relative motion between the components.

The FE model of C2–C7 segments was fixed at the inferior endplate of the C7 vertebra. The loading conditions consisted of a 75-N follower load applied to the odontoid of C2 vertebra to simulate the head weight, and a moment of 1.0-N·m producing either flexion, extension, lateral bending, or axial rotation (Lou et al., 2017; Rong et al., 2017; Wu et al., 2019). The ALL, PLL, nucleus pulposus, and annulus fibrosus were resected at C5/6, while the bilateral structures such as uncinate processes were preserved according to the real surgical procedure. The range of motion (ROM) was defined as the rotation from the neutral position to the end position at the load of 1.0-N·m. The ROM for each level was calculated based on the relative motions of the markers of each vertebra in each motion mode (Panjabi et al., 2001). The ROM of each segment of the intact cervical spine model under all moments was compared with previously published data to validate the model. The stress distribution of the interbody cage and two screws were tested under all experimental moments. The meshing sensitivity analysis was performed, with the element size ranging from 0.1 to 1.0 mm.

## Results

### Validation of the Developed FE Model

**FIGURE 2:**
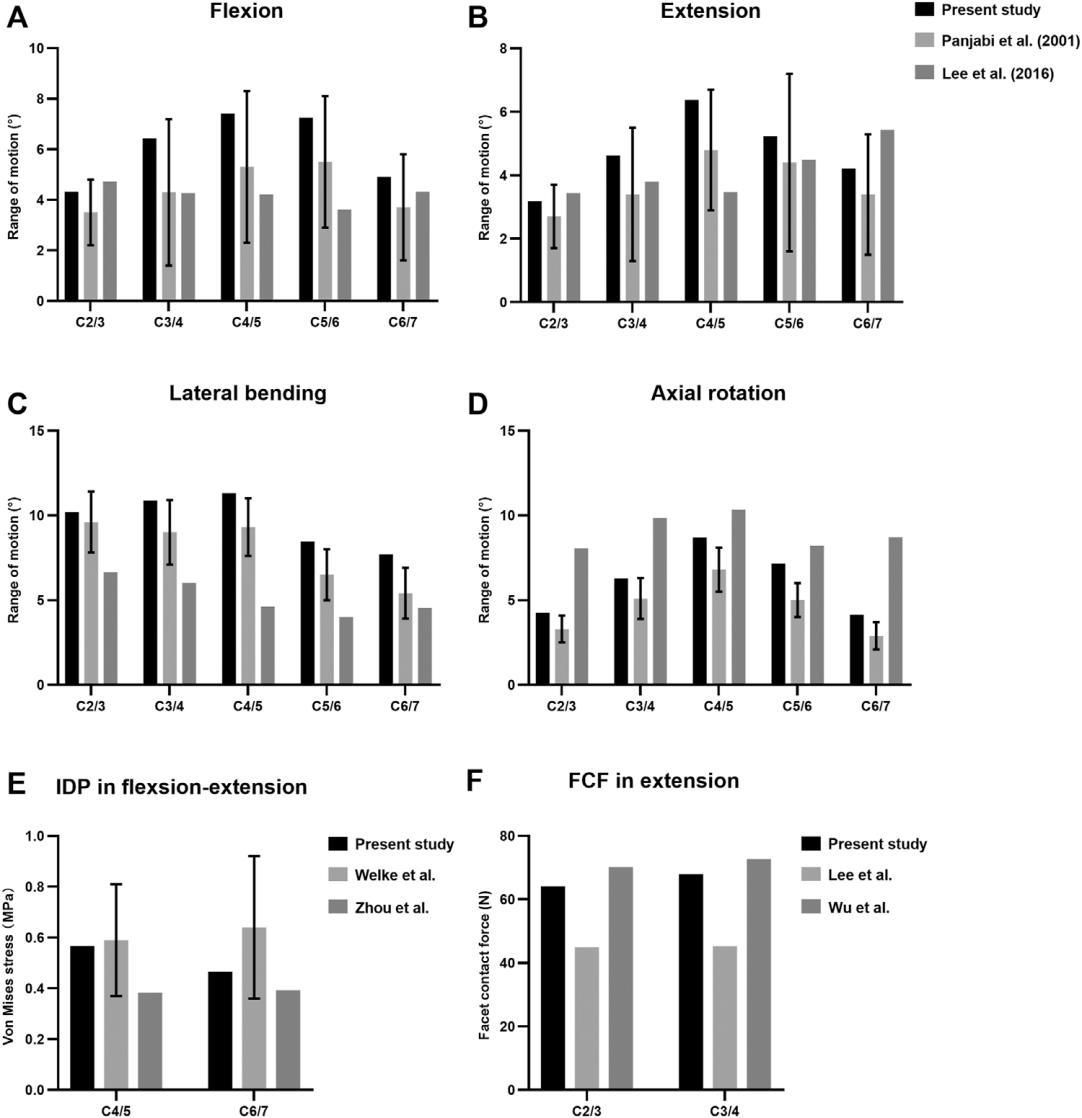
Comparison of the predicted ranges of motion (ROMs) **(A–D)**, intradiscal pressure (IDP) **(E)** and facet contact forces (FCF) **(F)** with published literature.

The predicted ROM of each segment in the intact cervical spine model was compared with previous published literature to assess the validity (Panjabi et al., 2001; Lee et al., 2016) (Figure 2). The values of lateral bending and axial rotation correspond to summating left and right angular motions. The overall ROMs of flexion, extension, lateral bending, and axial rotation were 30.33°, 23.62°, 48.56°, and 30.53°, respectively. The present segmental ROMs were within the range of the results from previous experiments studies. The maximal intradiscal pressure of adjacent levels was consistent with *in vitro* experiments and previous finite element results (Welke et al., 2016; Zhou et al., 2021), and the facet contact force (FCF) of the model was also well in agreement with the literature (Lee et al., 2011; Wu et al., 2019).

### Intradiscal Pressure and Facet Contact Force

**FIGURE 3:**
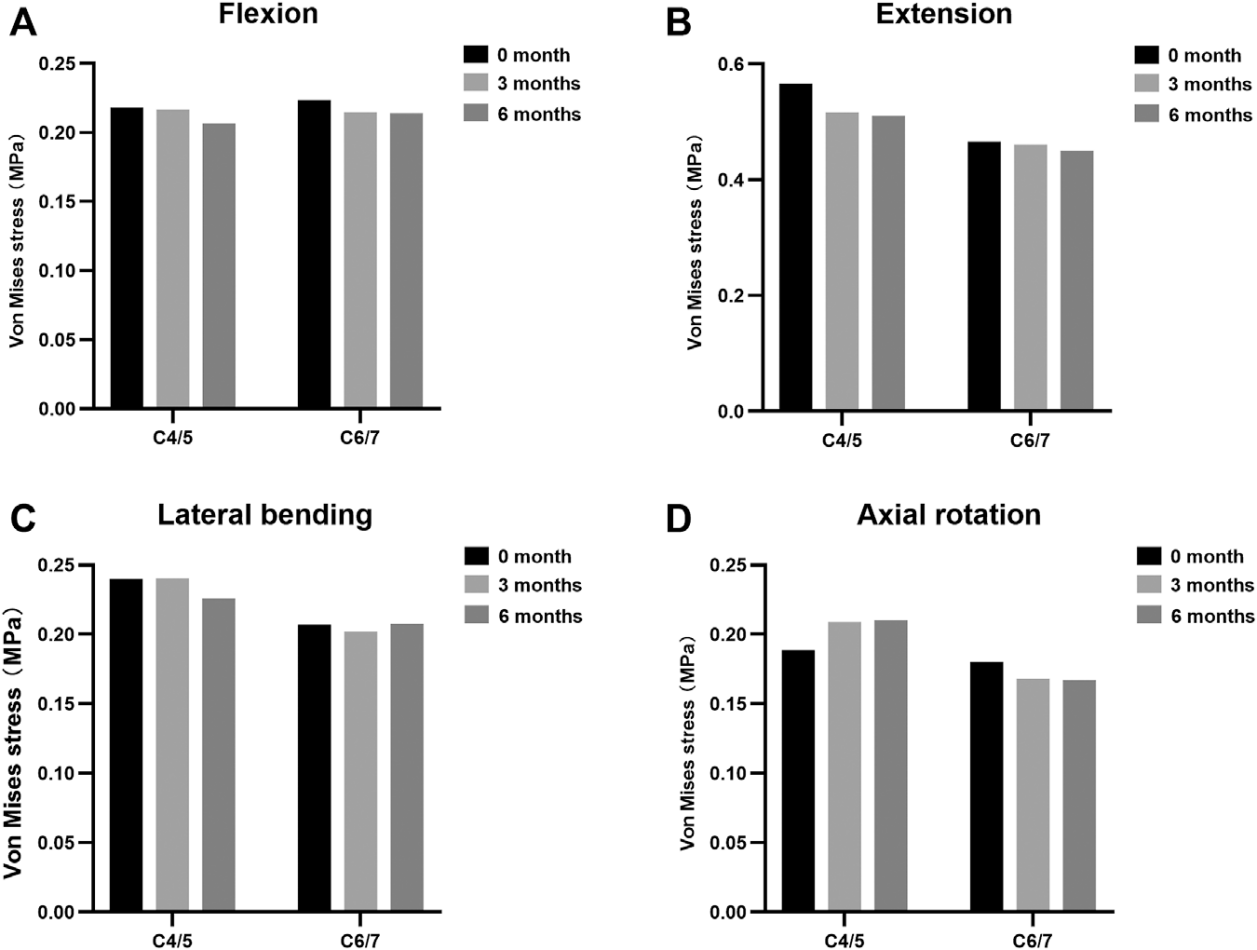
Maximal Von Mises stress at adjacent levels during different fusion courses in **(A)** flexion, **(B)** extension, **(C)** lateral bending, and **(D)** axial rotation.

**FIGURE 4:**
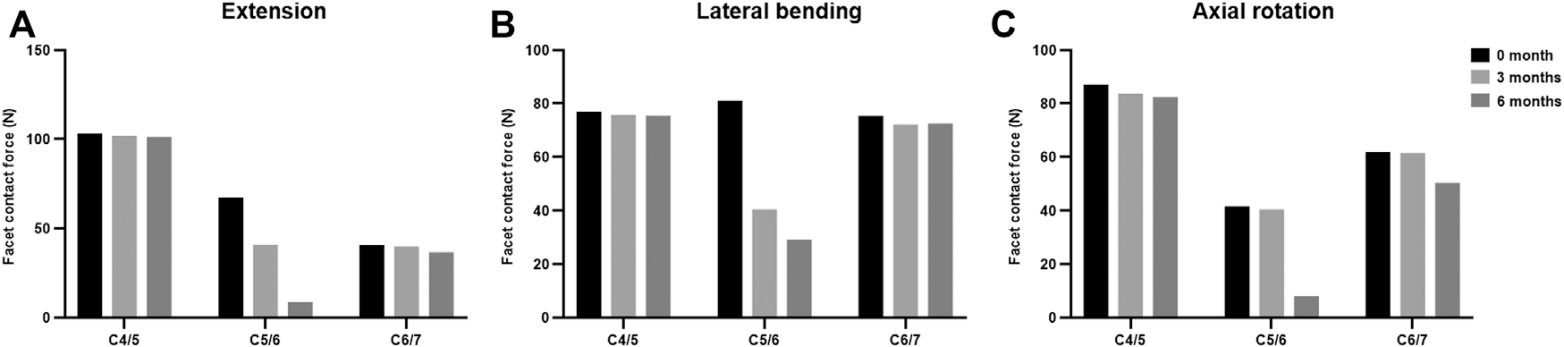
The facet contact force at the surgical and adjacent levels at different fusion stages in **(A)** extension, **(B)** lateral bending, and **(C)** axial rotation.

The maximal Von Mises stress on adjacent intervertebral disc in cervical motions are shown in Figure 3. The intradiscal pressure of C4/5 and C6/7 slightly changed at the incomplete and complete fusion stages compared with immediate post-operation. The maximal FCF in extension, lateral bending, and axial torsion is suggested in Figure 4. Compared to the FE model immediately after surgery, a stepwise reduction of maximal FCF was noted at the surgical segment (C5/6) at 3 and 6 months postoperatively. The FCF at C5/6 of the 3- and 6-month models decreased by 39.2% and 86.8% in extension, 49.9% and 63.9% in lateral bending, and 2.4% and 80.4% in axial rotation, respectively. The FCF of the adjacent segments (C4/5 and C6/7) barely changed.

### Stress Distribution

**FIGURE 5:**
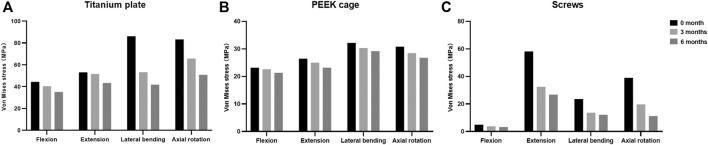
Maximal Von Mises stress at the surfaces of **(A)** titanium plate, **(B)** PEEK cage, and **(C)** screws at different fusion stages.

**FIGURE 6:**
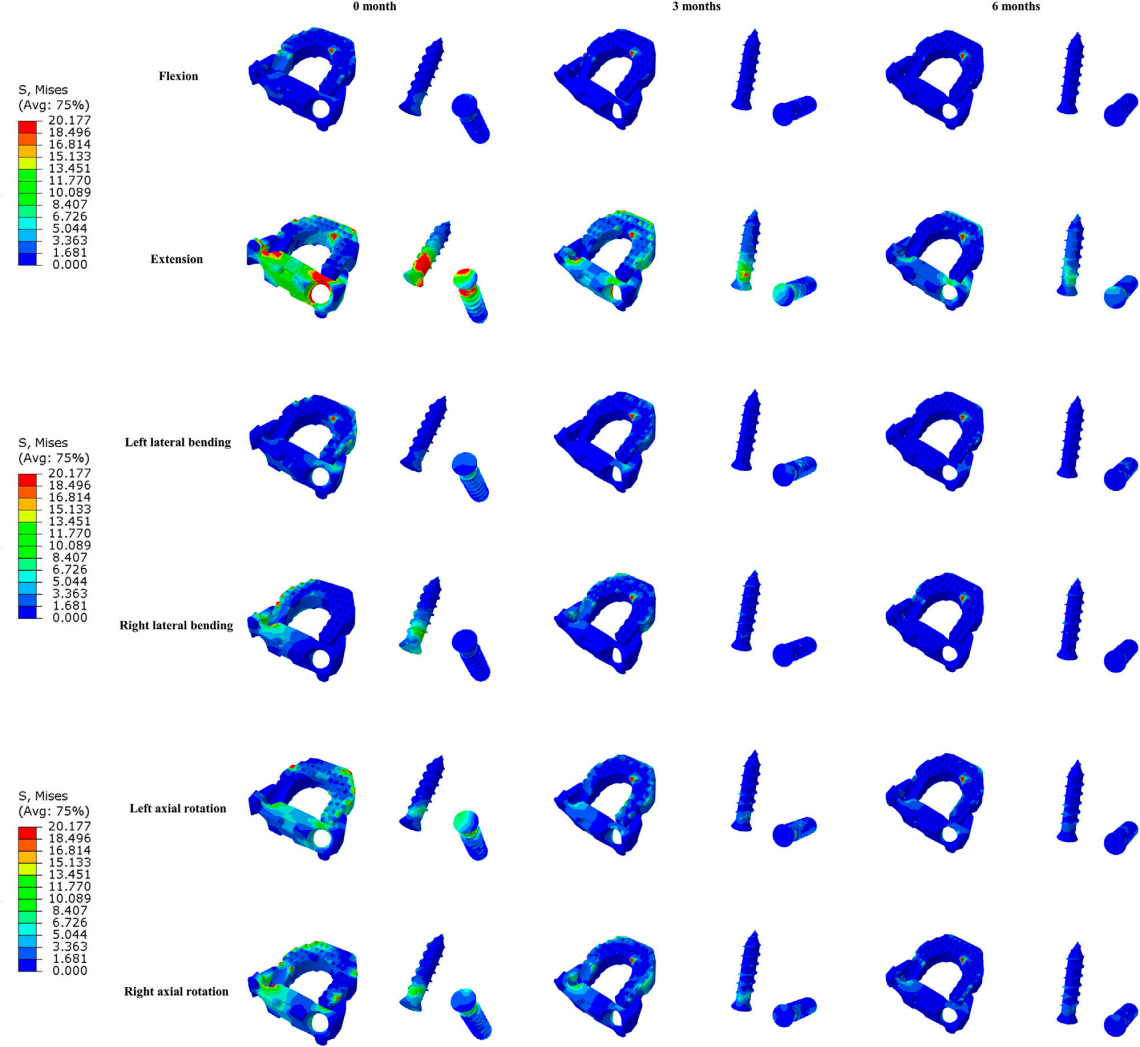
The Von Mises stress distribution on the surfaces of interbody fusion cage and screws at different fusion stages in all loading conditions.

The maximal Von Mises stress on the titanium plate, the PEEK cage, and screws are shown (Figure 5). The maximal stress on the surface of titanium plate of the 3-month model and 6-month model decreased by 9.1% and 20.8% in flexion, 2.8% and 18.5% in extension, 38.2% and 51.5% in lateral bending, and 21.0% and 38.9% in axial rotation, respectively, in comparison with the immediately postoperative model. The maximal stress on the surface of PEEK cage in 3 and 6 months decreased by 2.4% and 8.0% in flexion, 5.6% and 12.4% in extension, 5.8% and 9.4% in lateral bending, and 7.7% and 13.1% in axial rotation, respectively. Likewise, compared to the model immediately after surgery, the maximal stress on the screws of the 3- and 6-month models decreased by 23.7% and 34.6% in flexion, 44.2% and 53.9% in extension, 42.2% and 48.5% in lateral bending, and 49.4% and 71.2% in axial rotation, respectively. The stress distribution on the surface of interbody implant and screws in flexion, extension, lateral bending, and axial rotation is presented (Figure 6). In the model immediately after surgery, the stress unevenly distributed on the implant and screws. The stress distribution changed markedly in extension among three models of different fusion stages. When the interbody fusion was achieved (6-month model), the stress was more uniformly distributed in the interbody cage and screws in all loading conditions.

## Discussion

The purpose of this study was to investigate the biomechanics of adjacent intervertebral disc, facet joints, and instrumentation during the fusion process and explore the feasibility of reflecting the fusion status after ACDF through the load changes borne by the interbody fusion cage. Through comparison and analysis of the biomechanical changes of the three ACDF models of different fusion stages, we found that the stress was concentrated on the interbody cage immediately after surgery. When the adjacent vertebrae were completely fused, the stress on the implant surface reduced significantly and the stress pattern became more uniform. During the fusion process, the intradiscal pressure and FCF of adjacent segments changed slightly while the FCF of surgical segment showed a stepwise decrease.

ACDF is a procedure frequently indicated in the surgical management of cervical myelopathy or radiculopathy due to its demonstrated clinical outcomes (Zhao et al., 2020). A solid intervertebral fusion plays a key role in achieving satisfactory long-term outcomes. However, the current radiographic techniques to determine the degree of fusion are inaccurate and radiative (Walker, 1989; Brodsky et al., 1991; Blumenthal and Gill, 1993). The present study compared the Von Mises stress on the interbody cage in three C2–C7 ACDF models with different fusion statuses and found that the stress could potentially serve as the viable parameters to assess the fusion process after ACDF as an alternative of conventional static imaging methods. The novel method is based on the load transmission during bone healing. Ferrara et al. (2007) and Ferrara et al. (2008) investigated the feasibility of biomechanical characterization as a strategy to determine fusion status after ACDF with anterior plate and autografts. They found an increase in pressure at the graft–endplate interface and a decrease in load transmission along the ventral plate after bone fusion biomechanically simulated by polymethylmethacrylate (PMMA) insertion (Ferrara et al., 2007). Further *in vivo* assessment using telemetric pressure transducers demonstrated an increased pressure at the bone graft interfaces in living goats during the early stages after surgery, which may correlate with early graft subsidence (Ferrara et al., 2008). Despite the absence of fusion in this preliminary study, their results emphasized the implications of pressure at bone graft interfaces and its relevance to the clinical setting.

The Von Mises stress is widely used in FE study to evaluate the orthopedic implant design and risks for subsidence and implant failure (Yu et al., 2016; Zhang et al., 2016; Liu et al., 2020; Wo et al., 2021). Besides, the stress distribution on the surface of the implant may reflect the load transfer of different implants and biomechanical environments (Yu et al., 2016). We postulated that the stress on the interbody cage was a sensitive parameter to reflect the bone healing, thereby mainly analyzing the changes of stress in this study. The results of the stress on the interbody cage at different fusion stages could provide reference for future *in vivo* experiments with microsensors. Klosterhoff et al. (2017) and Klosterhoff et al. (2020) engineered a wireless implantable sensor and longitudinally measure strain across a rodent femoral defect in real time during functional rehabilitation activities. They found that real-time strain magnitude during ambulation correlated with the status of bone healing (Klosterhoff et al., 2020). Therefore, similar approach in interbody cage with implantable microelectromechanical systems (MEMS) sensor may provide promising diagnostic potential to assess intervertebral fusion and reduce clinical reliance on current radiation-emitting imaging methods (Ledet et al., 2018). In addition, due to the variability of the cervical anatomy across patients, several studies established patient-specific FE models for better clinical applications (Laville et al., 2009; Nikkhoo et al., 2019; Nikkhoo et al., 2021a). Nikkhoo et al. (2019) developed and validated a geometrically personalized FE model of the lower cervical spine based on parameters extracted from radiographs of each subject. They further analyzed the biomechanical effect of laminectomy using this parametric personalized FE model (Nikkhoo et al., 2021a). Laville et al. (2009) developed a parametric and subject-specific FE model of lower cervical spine and emphasized the role of geometry. Thus, biomechanical analysis of different fusion status using FE models with patient-specific parameters is warranted in further investigation.

In addition to the stress changes, the stress distribution of the implant interface may also be an important index reflecting the fusion status, particularly during daily movement. The present study found that the stress distribution on the surface of implant changed markedly and tended to be uniform during fusion, especially in extension. Likewise, in the FE simulations with four different stages image data postoperatively of a male amputee, Xu et al. (2016) investigated the relationship between the bone remodeling and the strain re-distribution around the *trans*-femoral osseointegrated implant system. The results revealed that the decrease in bone thickness around the distal end of implant resulted in an increase in strain while the bone growth in the proximal region led to a decrease in strain within this region. In the present study, the titanium plate with two self-locking screws in the Zero-P VA system functions as the conventional anterior cervical plate with the advantage of reducing irritation to the anterior soft tissue. Previous cadaveric studies of cervical spine have suggested that the application of anterior cervical plate could reverse the graft loads and excessively loads the bone graft in extension, but unloads the graft in flexion (Foley et al., 1999; DiAngelo et al., 2000). Peterson et al. (2018) used a validated custom force-sensing interbody implant to measure interbody loads in a sheep ACDF model in real time during fusion. They found that the average change in interbody force magnitude from full extension to full flexion decreased significantly during fusion. Furthermore, Park and Jin (2019) and Jin and Park (2021) simulated extragraft bone formation at the C5–C6 motion segment after ACDF using a developed finite element model and a sequential bone-remodeling algorithm in flexion and extension. The results suggested that both the stress and strain energy density slightly changed in flexion while a significantly stepwise decrease was predicted in extension until the fusion was terminated (Park and Jin, 2019). Therefore, more attention should be paid to the stress distribution of interbody implant in extension because of the significant changes during fusion.

Regarding the FCF during fusion, our findings suggested that the FCF of the surgical segment was significantly reduced with the development of intervertebral fusion, indicating the load transmission process during fusion. It has been reported that 64% of the axial load is handled by the posterior column of cervical spine while 36% is supported by anterior column in physiological condition (Pal and Sherk, 1988). Hence, the intervertebral fusion in the anterior column may have shared partial axial load that was originally carried by posterior column, resulting in the decrease of FCF. In an *in vivo* sheep model of anterior lumbar interbody arthrodesis, Takahata et al. (2005) analyzed the change of load distribution through fusion mass and spinal instrumentation in the healing process. They found that the formation of bridging trabeculated bone across the fused segment resulted in increased load transmission through the anterior fusion mass accompanied by decreases in the loads of rod instrumentation system and posterior spinal elements. In addition, Kanayama et al. (1997) established a posterolateral spinal arthrodesis sheep model and measured the strain on the spinal instrumentation using rods instrumented with strain gauges throughout the healing process. They demonstrated that the load-sharing properties of spinal instrumentation decreased concurrently with the development of the spinal fusion. In the early period after arthrodesis, the load across the surgical segment was mainly borne by the rods and screws; when the fusion was completed, the intervertebral load was primarily distributed to the solid fusion mass, resulting in the unloading condition of the hardware. Therefore, there may also be load changes on the instrumentation during fusion after posterior cervical surgery, which requires further research.

Increased intradiscal pressure of adjacent segments of surgical segments after ACDF may be related to the development of adjacent segment degeneration (ASD) (Eck et al., 2002). In the present study, we found that there were no significant changes in intradiscal pressure of adjacent segments at different fusion stages after ACDF, indicating that biomechanical changes in adjacent segments may be mainly due to the instrumentation rather than bony fusion. Of note, a zero-profile cage was used in the present ACDF surgery, whereas the biomechanical effect of the zero-profile cage and the cage plus plate on the adjacent segments may be different. Li et al. (2020) found that the intradiscal pressure of adjacent segments in the plate-interbody model was slightly larger than that in the zero-profile model after single-level ACDF in a FE study. Hua et al.(2020a) found that the intradiscal pressure was increased at adjacent segments using either a zero-profile device or cage plus plate while the loss of ROM at the fused segment was larger in cage plus plate group. Guo et al. (2021) found that the different plate-to-disc distance had no effect on adjacent intradiscal pressure following single-level ACDF, while it may affect the stress of bone graft and titanium plate. The intervertebral reconstructive height may also influence the intradiscal pressure of adjacent segments (Zhou et al., 2021). Thus, further study is needed to analyze the biomechanical effect of interbody fusion device with different structures during fusion. In addition, the material properties of intervertebral discs in FE models may also affect the biomechanical results. The hyperelastic material properties were used in the intervertebral disc modeling in the studies of Nikkhoo et al. (2019) and Gandhi et al. (2019). Kandil et al. (2019) and Kandil et al. (2021) constructed a lumbar disc geometry, and the properties of disc annulus fibrosis were described using a microstructure-based chemo-viscoelastic model. Recently, Li et al. (2021) established a poroelastic FE model of cervical spine to determine the effect of degenerative status of adjacent disc on its biomechanics after single-level ACDF. They found that the stress in pre-existing degenerated disc experienced was larger than that in normal situation. Khalaf and Nikkhoo (2021) and Nikkhoo et al. (2021b) developed a geometrically patient-specific FE model of the lumbosacral spine for comparison of the spinal biomechanics with different fixation devices, in which poro-hyperelastic materials were used for the intervertebral discs modeling. Therefore, it may be more realistic to analyze the biomechanics of adjacent intervertebral discs by means of these advanced modeling and further investigation is warranted.

There are several limitations in this study. First, simplified parameters such as material properties, boundary conditions, and frictionless contact cannot completely represent actual *in vivo* conditions after surgery. Only linear elastic materials were used for the cervical vertebral body and the intervertebral disc. Although they have partial effect on the biomechanical environment, the present study primarily focused on the changing trends during fusion process. The material properties should be noticed if the objective of the study changes. As discussed above, material properties such as the hyperelastic, viscoelastic, or poroelastic material within the intervertebral disc can result in better biomechanical predictions. A more realistic model needs to be developed in future studies. Second, the surrounding musculature was not constructed in this study to simplify the model, which may not accurately reflect the cervical spine movement during physiological loading paradigms and probably affect the implant load-sharing. Third, the position and size of the implants are likely to have variations caused by multiple factors such as the patient’s anatomical variation, surgical carpentry, and surgeon’s preference, possibly resulting in different stress distribution and changes during healing. The present study analyzed the biomechanics of only one type of interbody fusion device while the biomechanical changes after ACDF using cage plus plate or the interbody cage with different structures after ACDF may be different (Hua et al., 2020a; Zhou et al., 2021). Besides, other cervical fusion procedures, such as anterior cervical corpectomy and fusion and the posterior cervical fusion surgery, may have similar biomechanical trends to this study and deserve further analysis. Fourth, the geometry of human spine varies among individuals; however, the model was developed based on the data from a single patient, so the current modeling data should be interpreted with caution. Statistical comparison was not performed in this study because the results in FE analysis were obtained from mathematical calculations without standard deviation. Future studies using geometrically personalized models can be valuable for better clinical application (Nikkhoo et al., 2019). However, the main conclusions of the present study were based on the comparative analysis among three models, thereby being less influenced by the aforementioned simplifications. The biomechanical changes during fusion process after ACDF must be evaluated first with finite element analysis, and future *in vivo* experiments using interbody fusion device with microsensors are warranted to monitor the long-term, real-time load changes.

## Conclusion

In conclusion, this FE study analyzed the biomechanical changes of adjacent intervertebral disc, facet joints, and instrumentation during the fusion process, and explored the feasibility of reflecting the fusion status after ACDF by the stress changes on the interbody fusion cage. Through a follow-up study of 6 months, the stress on the surfaces of interbody implant and screws significantly decreased in all loading conditions. The stress distribution in extension markedly changed in the fusion process. After solid intervertebral fusion formed, the stress distribution on the surfaces of interbody cage and screws was more uniform. A stepwise decrease of facet contact force at the surgical segment was also noted as the fusion proceeded. Future studies are needed to develop the interbody fusion device with wireless sensors to achieve longitudinal real-time monitoring of the stress distribution during the course of fusion.

## Data Availability

The raw data supporting the conclusion of this article will be made available by the authors, without undue reservation.
